# Effects of Fatty Acids on Intracellular [Ca^2+^], Mitochondrial Uncoupling and Apoptosis in Rat Pachytene Spermatocytes and Round Spermatids

**DOI:** 10.1371/journal.pone.0158518

**Published:** 2016-07-18

**Authors:** Joaquín Paillamanque, Cristian Madrid, Emerson M. Carmona, Nelson Osses, Ricardo D. Moreno, Gerardo M. Oresti, José A. Pino, Juan G. Reyes

**Affiliations:** 1 Instituto de Química, Pontificia Universidad Católica de Valparaíso, Valparaíso, Chile; 2 Departamento de Fisiología, Pontificia Universidad Católica de Chile, Santiago, Chile; 3 Instituto de Investigaciones Bioquímicas de Bahía Blanca (INIBIBB), Bahia Blanca, Argentina; Cornell University, UNITED STATES

## Abstract

The aim of this work was to explore the ability of free arachidonic acid, palmitic acid and the unsaturated fatty acids oleic acid and docosahexaenoic acid to modify calcium homeostasis and mitochondrial function in rat pachytene spermatocytes and round spermatids. In contrast to palmitic acid, unsaturated fatty acids produced significant increases in intracellular calcium concentrations ([Ca^2+^]_i_) in both cell types. Increases were fatty acid specific, dose-dependent and different for each cell type. The arachidonic acid effects on [Ca^2+^]_i_ were higher in spermatids than in spermatocytes and persisted when residual extracellular Ca^2+^ was chelated by EGTA, indicating that the increase in [Ca^2+^]_i_ originated from release of intracellular calcium stores. At the concentrations required for these increases, unsaturated fatty acids produced no significant changes in the plasma membrane potential of or non-specific permeability in spermatogenic cells. For the case of arachidonic acid, the [Ca^2+^]_i_ increases were not caused by its metabolic conversion to eicosanoids or anandamide; thus we attribute this effect to the fatty acid itself. As estimated with fluorescent probes, unsaturated fatty acids did not affect the intracellular pH but were able to induce a progressive decrease in the mitochondrial membrane potential. The association of this decrease with reduced reactive oxygen species (ROS) production strongly suggests that unsaturated fatty acids induced mitochondrial uncoupling. This effect was stronger in spermatids than in spermatocytes. As a late event, arachidonic acid induced caspase 3 activation in a dose-dependent manner both in the absence and presence of external Ca^2+^. The concurrent but differential effects of unsaturated fatty acids on [Ca^2+^]_i_ and mitochondrial functions are additional manifestations of the metabolic changes that germ cells undergo during their differentiation.

## Introduction

The functional relationship between germ cells and Sertoli cells (SC) in the mammalian seminiferous tubules occurs either through juxtacrine signalling (adhesion molecules) or paracrine signalling (molecules secreted into the extracellular space of the adluminal compartment) [1]. However, the precise composition of the extracellular environment of germinal cells in the seminiferous tubules has not been determined. Glucose gains access to the luminal compartment through SC [2], which also secrete lactate to the luminal and adluminal compartments as a product of SC glucose metabolism [3–5]. Since the glycolytic activity of SC is stimulated by follicle stimulating hormone (FSH), β-adrenergic agonists, interleukin 1 β (IL1-β) and tumour necrosis factor α (TNF-α) [4–7], a similar, though inverse, regulation of lactate and glucose secretion toward the adluminal compartment is expected. Lactate and glucose have been shown to modulate [Ca^2+^]_i_, intracellular pH (pH_i_) and mechanisms of cell death in spermatogenic cells [8,9].

SC also produce arachidonic acid (AA) and some of its metabolites in an FSH-regulated manner [10]. In a mouse SC-derived cell line (TM4), activation of CD95 (Fas) [11,12] activates cytosolic phospholipase A2 (PLA2) and AA release [13]. Likely via an autocrine loop, AA activates glycolytic lactate production in SC [14]. Accumulated evidence strongly suggests that AA is part of the regulatory signalling networks regulating spermatogenesis. In support of this idea, a diet rich in AA has a large impact on animal fertility [15] and mice lacking the group VI PLA2 isoform show impaired sperm motility and greatly reduced fertility [16].

Unsaturated fatty acids (UFAs) are known to mobilize [Ca^2+^]_i_ in pancreatic β cells and neutrophils [17,18]. The importance of [Ca^2+^]_i_ homeostasis in spermatogenic progression is strongly suggested by the fact that a blockade of ryanodine receptors reduces spermatogonia proliferation and induces meiosis in spermatocytes [19]. Additional evidence in support of a role for [Ca^2+^]_i_ mobilization in germ cells is that 1) Ca^2+^ entry is involved in expression of the antiapoptotic proteins Bcl-xS and Bcl-xL [20]; 2) mice deficient in CIB1, a calcium binding protein, show increased apoptosis in spermatogenic cells [21]; 3) voltage-activated calcium channel blockers induce arrest of spermatogenesis at the elongating spermatid level in mice [22] and 4) genetic ablation of CAMK4 (calmodulin dependent kinase) impairs spermatogenesis [23]. The aim of this work was to examine whether free fatty acids regulate [Ca^2+^]_i_ homeostasis of pachytene spermatocytes and round spermatids. We chose two polyunsaturated FA, AA and docosahexaenoic acids (DHA) as well as oleic acid (OA) and palmitic acid (PA) because they are abundant in membrane glycerophospholipids of all cells in the seminiferous epithelium [24,25].

## Materials and Methods

### Animals

Adult (40–60 days old) male Sprague–Dawley rats were acquired from the Animal Facility in the Faculty of Biological Sciencies Pontifical Catholic University of Chile. The rats were housed under a 12L:12D cycle with water and rat chow *ad libitum* and were euthanized by narcotization with CO_2_ followed by cervical dislocation.

### Ethical statement

All the experiments were conducted in accordance with the guidelines outlined by the Consortium for Development in the Guide for the Care and Use of Agricultural Animals in Agricultural Research and Teaching and by the National Research Council. All experimental protocols were reviewed and approved by the Chilean National Fund for Science and Technology (FONDECYT) and the Ethics Committee of the Pontificia Universidad Catolica de Valparaíso (EC-PUCV-10/2013). None of the authors served on this committee.

### Chemicals

Fura-2 acetoxymethyl ester; 2',7'-bis-(2-carboxyethyl)-5-(and-6)-carboxyfluorescein acetoxymethyl ester (BCECF); bis-(1,3-dibutylbarbituric acid) trimethine oxonol (oxonol), 2',7'-dichlorodihydrofluorescein diacetate (H2DCFDA; reactive oxygen and nitrogen species (ROS/RNS) sensor) and 5,5′,6,6′-Tetrachloro-1,1′,3,3′-tetraethyl-imidacarbocyanine iodide (JC-1), were purchased from Molecular Probes (Life Technologies, USA). Arachidonic, oleic, docosahexanoic, palmitic acid, indomethacin (inhibitor of cyclooxygenase, Indomet), nordihydroguaiaretic acid (inhibitor of lipoxygenase, NDGA), valinomycin, luciferin, luciferase, rhodamine 123, salts and buffers were obtained from Sigma-Aldrich (St. Louis, MO, USA). 2-(1-thienyl)ethyl 3,4-dihydroxybenzylidenecyanoacetate (inhibitor of lipoxygenase, 2-TEDC), was purchased from Tocris Bioscience (through Gene-Xpress, Chile).

Indomethacin, NDGA and 2-TEDC have been shown to be cell permeant and have 50% inhibitory concentrations of 0.1–0.2 μM for indomethacin [26], 0.5–1.0 μM for NDGA [27] and 0.09, 0.013 and 0.5 μM (5-, 12-, and 15-lipoxygenases, respectively) for 2-TEDC [28].

### Experimental procedure

Adult rats were maintained in groups of three to four animals per cage. Groups of two to three rats were chosen at random and used to isolate pachytene spermatocytes and round spermatids. Rats were euthanized in the Animal Facility, and the testes of each rat were isolated in a room specially equipped for this procedure.

### Rat pachytene spermatocytes and round spermatid isolation

Rat spermatogenic cell populations were isolated using velocity sedimentation separation through a 2–4% bovine serum albumin (BSA) gradient, as described [29]. The pachytene spermatocyte (85 ± 5% purity) and round spermatid fractions (92 ± 4% purity) were identified both by their size as well as by the typical nuclear feature after staining with Hoechst 33342 [30]. Unless otherwise stated, measurements of cell suspensions of pachytene spermatocytes or round spermatids labelled with specific fluorescent probes were collected using a Fluoromax 2 fluorimeter (Jobin-Yvon-Spex, NJ, USA).

### [Ca^2+^]_i_ measurements of rat spermatogenic cells in suspension

Rat spermatogenic cells were resuspended (20 x 10^6^ cells/mL) in KH media (140 mM NaCl, 4 mM KCl, 1.6 mM MgCl_2,_ 1.6 mM KH_2_PO_4_, 10 mM HEPES, pH 7.4) containing or lacking Ca^2+^ (0.5 mM CaCl_2_ or 1 mM EGTA, respectively). Metabolic substrates for the cells were either 5 mM L-Lactate (KH-lactate) or 5 mM glucose (KH-glucose). The cells in KH-lactate media were loaded with 5 μM of the calcium probe Fura-2 AM by incubation for 1 hr at room temperature in an oxygenated atmosphere followed by three washes at 4°C and resuspension in the same media The fluorescence measurements were performed by adding a concentrated cell suspension (50 μL) to a temperature-regulated spectrofluorimeter cuvette (3.0 mL, with stirring) containing 2.5 mL of one of the four different types of media described above to which fatty acids have previously been added. The [Ca^2+^]_i_ determinations were carried out using a ratiometric method as described [31]. Fura-2 calibration was performed by cell lysis with digitonin (20–25 μg/mL) and addition of 1 mM EGTA to determine F_min_ or addition of 3 mM CaCl_2_ to determine F_max_.

Measurements described in this work were made without the addition of external Ca^2+^ and 1 mM EGTA, unless otherwise indicated. The initial rates of [Ca^2+^]i changes induced by FAs were estimated by linear regression of the [Ca^2+^]i values taken between 10s and 110 s after FAs addition.

### Viability assays

The percentage of cells maintaining membrane integrity was visualized using a permeant nuclear fluorescent probe (Hoechst 33342) and a non-permeable probe (propidium iodide, PI). As a second criterion, the release of a cytosolic enzyme (lactate dehydrogenase, LDH) was quantified as the decrease in NADH concentration after addition of the spermatogenic cell extracellular medium to a solution buffered to pH 7.5 with 20 mM Tris-HCl and containing 0.2 mM NADH and 5 mM pyruvate.

### Mitochondrial membrane potential changes measured with rhodamine 123 fluorescence

The mitochondrial membrane potential changes induced by FA were measured using the lipophilic, cationic probe rhodamine 123 (R123). Potential-dependent accumulation of this probe in energized mitochondria results in a relatively weak fluorescence signal due to self-quenching. Dissipation of the normally negative mitochondrial membrane potential (e.g., by an uncoupler) is marked by an increase in fluorescence due to distribution of the dye throughout the cell, which results in a green shift in the excitation spectra. The fluorescence with excitation ratio 520/490 and an emission wavelength of 530 nm reflects the mitochondrial membrane potential and decreases upon depolarization of the membrane [32,33]. Spermatogenic cells were resuspended (2 x10^6^ cells/mL) in High K^+^-media (HK^+^) that contained no nominal calcium (5 NaCl mM, 140 KCl mM, 1.5 mM MgCl_2_, 10 mM KH_2_PO_4_, 10 mM Hepes, 1 mM EGTA, 0.2 mM EDTA, pH 7.4; supplemented with 10 mM lactate). Membranes were permeabilized with digitonin using 20 and 25 μg/mL for spermatids and pachytene spermatocytes, respectively [34], and exposed to 300 nM of R123 for 5 min. Subsequently, the excitation ratio (520/490 nm) at an emission wavelength of 530 nm was measured at 33°C as a function of time.

The JC-1 dye was also used to evaluate FA-induced mitochondrial membrane potential changes. Accumulation of this probe in active mitochondria is sensitive to the membrane potential, and the fluorescence emission shifts from green to red due to the formation of red fluorescent J-aggregates. Mitochondrial depolarization is indicated by a decrease in the red/green fluorescence intensity ratio. The emission ratio 590/560 with an excitation wavelength of 514 nm reflects the mitochondrial membrane potential and is expected to decrease upon depolarization [35]. For fluorimetric measurements, 200 nM JC-1 was incubated for 15 min with spermatogenic cells resuspended (1 x 10^6^ cells/mL) in KH-lactate media supplemented with 0.5 mM CaCl_2_. Subsequently, vehicle (< 2 μL ethanol) or the fatty acids were added.

### Membrane potential measurements using bis-(l,3 diethyl)thiobarbiturate trimethine oxonol

Changes in plasma membrane potential were determined using the negatively charged, permeant fluorescent probe oxonol with an excitation at 535 nm and an emission at 560 nm. After measuring the baseline fluorescence of 500 nM oxonol in a 3 mL cuvette kept at 33°C, 50 μL of a concentrated cell suspension was added to reach a final cell concentration 2 x 10^6^ cells/mL. After stabilization of the fluorescence at 33°C, the FAs were added to the cuvette to the specified final concentrations and fluorescence levels were allowed to equilibrate. Finally, fluorescence was measured following the addition of valinomycin at a final concentration of 0.5 μM, which has been previously shown to hyperpolarize round spermatids [36]. In the absence of cells, changes in oxonol fluorescence were not observed for any of the FA concentrations examined in this study.

### Intracellular pH measurements

The pH_i_ of spermatogenic cells was estimated from fluorescence levels of intracellular BCECF. Cells at a density of 2 x 10^6^ cells/mL were loaded with BCECF by incubation in KH-Ca^2+^-lactate media with 2 μM acetoxymethy1 BCECF for 30 min at 33°C in 95% O_2_/5% CO_2_. The measurements were performed in a Fluoromax-2 fluorometer (Jobin Ivon-Spex, USA) at an excitation fluorescence ratio of 505/445 and a 530 nm emission wavelength. Observation of the cells by epifluorescence microscopy showed a homogeneous distribution of the dye throughout the cytoplasm.

Intracellular BCECF was calibrated in HK+ media with 1 μM nigericin by measuring the excitation fluorescence ratio of 505/445 nm at different extracellular pH values [37]. The pH of the extracellular saline was varied between pH 5.5 and 8.0. Fluorescence ratios were obtained at each calibration pH and a standard curve was generated using Microcal Origin version 7.0 (Microcal Software, Inc., MA, USA). Intracellular pH values were calculated from the experimental fluorescence ratios obtained before and after addition of FA to the cells.

### ATP release assay

The release of ATP from pachytene spermatocytes or round spermatids into the extracellular milieu was estimated using a luciferin-luciferase assay [38]. Measurements were taken with the fluorimeter lamp off, the excitation shutter closed, a wide emission slit width centred at 550 nm and 4 s integration time. Successive additions of cells, luciferin, luciferase, AA and digitonin were performed to test for intrinsic cell luminescence, possible residual ATP in the luciferin-luciferase reagent and the effect of AA. Release of cellular ATP upon addition of digitonin was measured as a positive control.

### ROS/RNS production measurements

Rat spermatogenic cells were resuspended in KH-Ca^2+^-lactate media to a final concentration of 6 x 10^5^ cells/mL. Cells were pre-incubated for 3 min at 33°C and then H_2_DCFDA (2.5 μM final concentration) was added to the cell suspension. Fluorescence measurements with an excitation at 495 nm and emission at 529 nm were made of dichlorofluorescein (DCF) (which is the product of H_2_DCFDA oxidation) at 15 s intervals for 15 min in an Appliskan multiwell fluorescence plate reader (Thermo Fisher Scientific, Inc.). Standardization of the probe and data analysis was performed as described by [39].

### Caspase 3 activity measurements

Pachytene spermatocytes or round spermatids were prepared at 1.5–2 x 10^6^ cells/mL in KH-Ca^2+^-lactate media. Each well of a 96 multi-well plate received 200 μL of the resulting cell suspension. To each well, either the oxidative stress and apoptosis inducer *tert*-butyl hydroperoxide (tBHP, 1 mM stock in DMSO) was added to a final concentration of 200 μM or AA (1 mM stock in ethanol) was added to different final concentrations and homogenized in the wells. Finally, the caspase-3 fluorogenic substrate IV (#264150, Calbiochem-Merck Millipore, Chile) was added to a final concentration of 30 μM (5 mM stock in DMSO). Measurements with an excitation at 430 nm and an emission at 480 nm were performed at 10 min intervals for 240 min.

### Data and statistical analysis

The initial rate of FA-induced Ca^2+^ entry was determined by linear regression of the [Ca^2+^]_i_ vs. time data between 30 and 200 s after FA addition. Statistical differences between treatment and control rates of increase in [Ca^2+^]_i_ were analysed by applying a t-test with Wellch’s correction or an ANOVA followed by a post-test when different experimental conditions were compared. When the data was expressed as a percentage increase in [Ca^2+^]_i_ by each FA or when the F-test indicated significant differences in group variances, the comparison between treatment and control was performed using non-parametrical Mann-Whitney analysis [40]. In all cases, measurements were carried out from samples derived from at least two cell preparations with four testicles each (two rats). The results were expressed as the mean ± standard error of the mean (SEM).

## Results

### Dose-response curves of [Ca^2+^]_i_ changes induced by FA in spermatogenic cells

**Fig 1:**
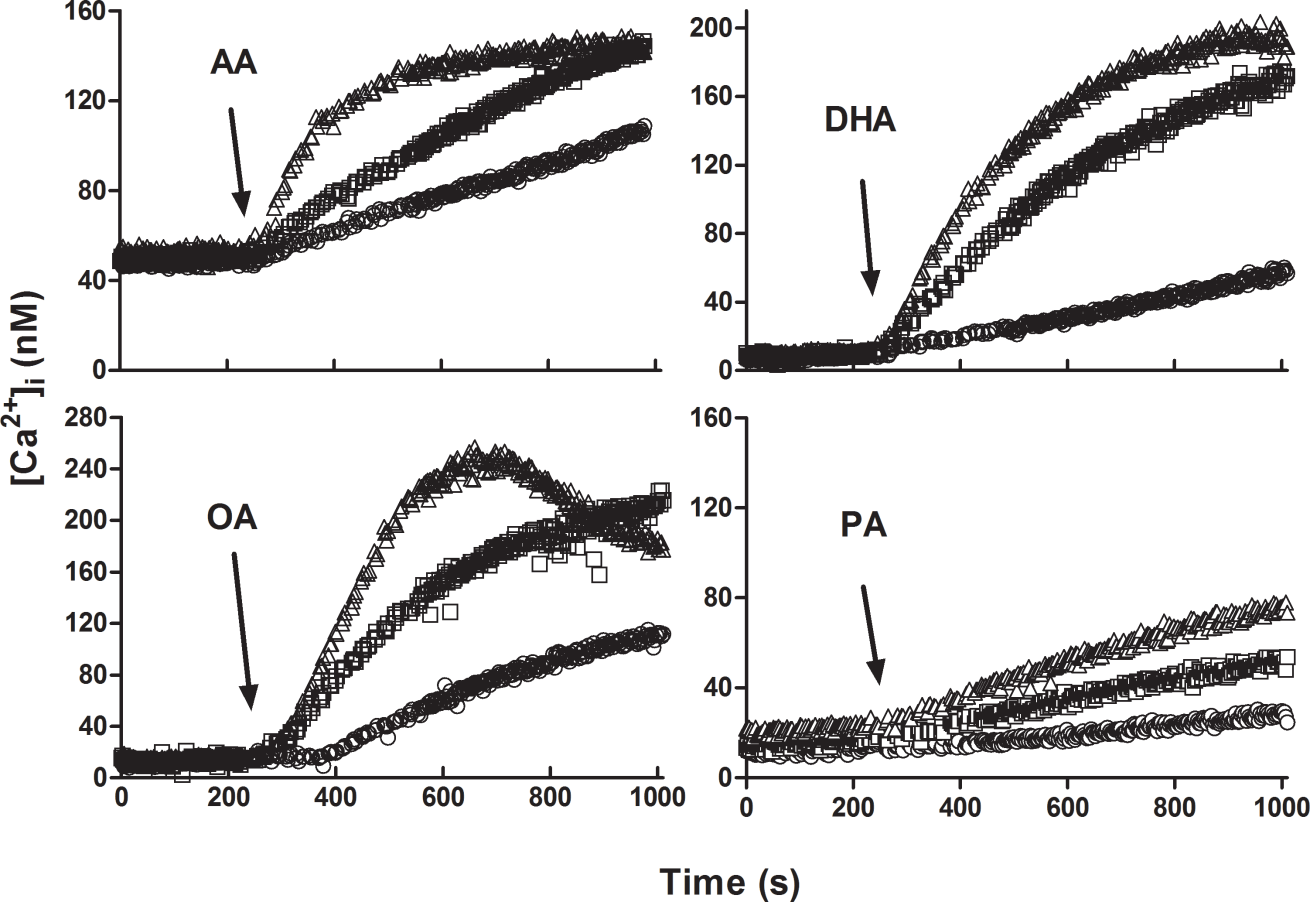
Time course of FA-induced [Ca^2+^]_i_ changes in round spermatids in the nominal absence of external Ca^2+^. After loading the cells with Fura-2, 2 (○), 4 (□) or 8 μM (Δ) of each fatty acid (AA, DHA, OA and PA) was added to a suspension of round spermatids in KH-lactate-EGTA at the times indicated by arrows.

**Fig 2:**
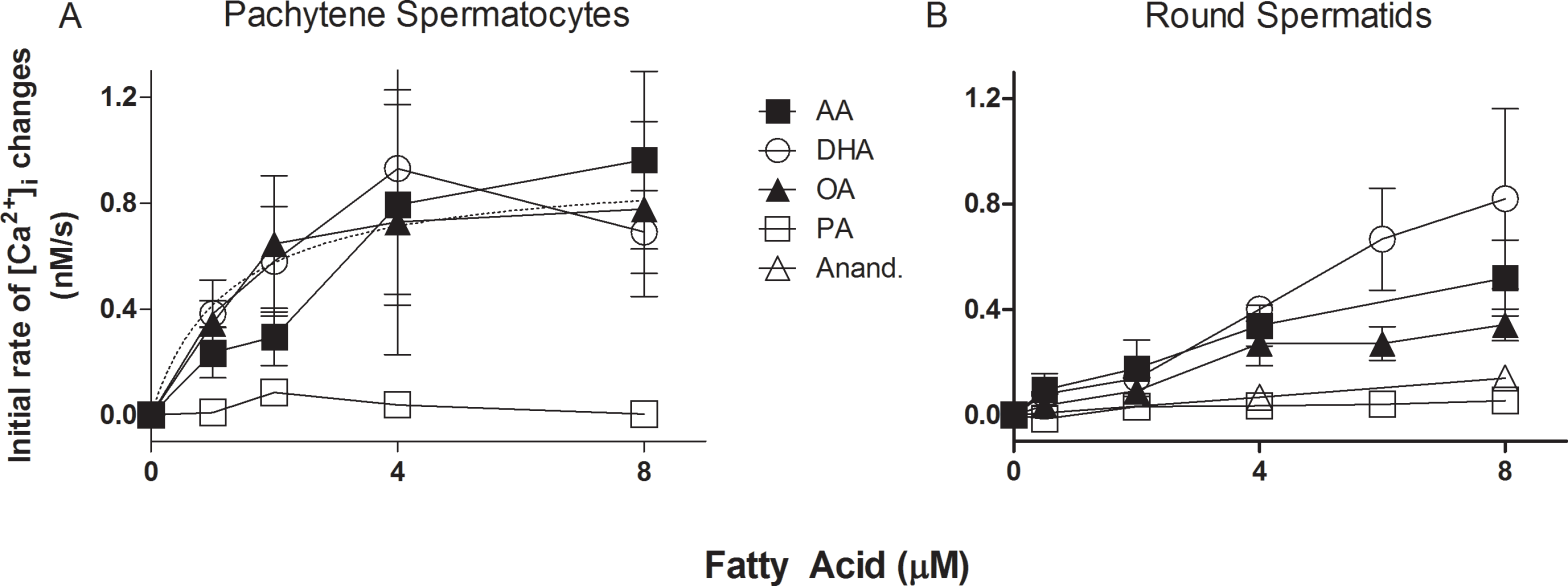
Concentration dependence of AA-induced changes in [Ca^2+^]_i_. **(A)** Dose response curves for the rate of [Ca^2+^]_i_ changes (nM/s) at different AA, DHA, OA, and PA total concentrations in suspensions of pachytene spermatocytes. The dotted line represents a hyperbolic fit of the DHA data. **(B)** Dose response curves for the rate of [Ca^2+^]_i_ changes at different AA, DHA, OA, and PA total concentrations in suspensions of round spermatids. Similar concentrations of anandamide were included for comparison. These measurements were performed in the absence of external Ca^2+^.

Fig 1 shows the kinetics of [Ca^2+^]_i_ after addition of arachidonic (AA), docosahexaenoic (DHA), oleic (OA) and palmitic acid (PA) in round spermatids. [Ca^2+^]_i_ changes induced by FA in KH-lactate-EGTA media were dose-dependent. At 8 μM, OA showed a biphasic curve that was not studied further and could be interpreted either as Ca^2+^ reuptake into intracellular calcium stores (ICaS) or as Ca^2+^ exit from the cells after 700 s of OA addition. The rates of increase in [Ca^2+^]_i_ induced by different concentrations of AA, DHA, OA and PA in pachytene spermatocytes and round spermatids are compared in Fig 2. The three UFAs, but not PA, show a clear increase in [Ca^2+^]_i_ in pachytene spermatocytes (Fig 2A) and round spermatids (Fig 2B). An interesting difference between the [Ca^2+^]_i_ rise among these cell types was that spermatocytes showed a clearer trend toward saturation of the FA effect on [Ca^2+^]_i_, with a K_0.5_ of approximately 1.3 μM.

### External Ca^2+^ and metabolic substrate dependence of the AA-induced effects in spermatogenic cell [Ca^2+^]_i_

**Fig 3:**
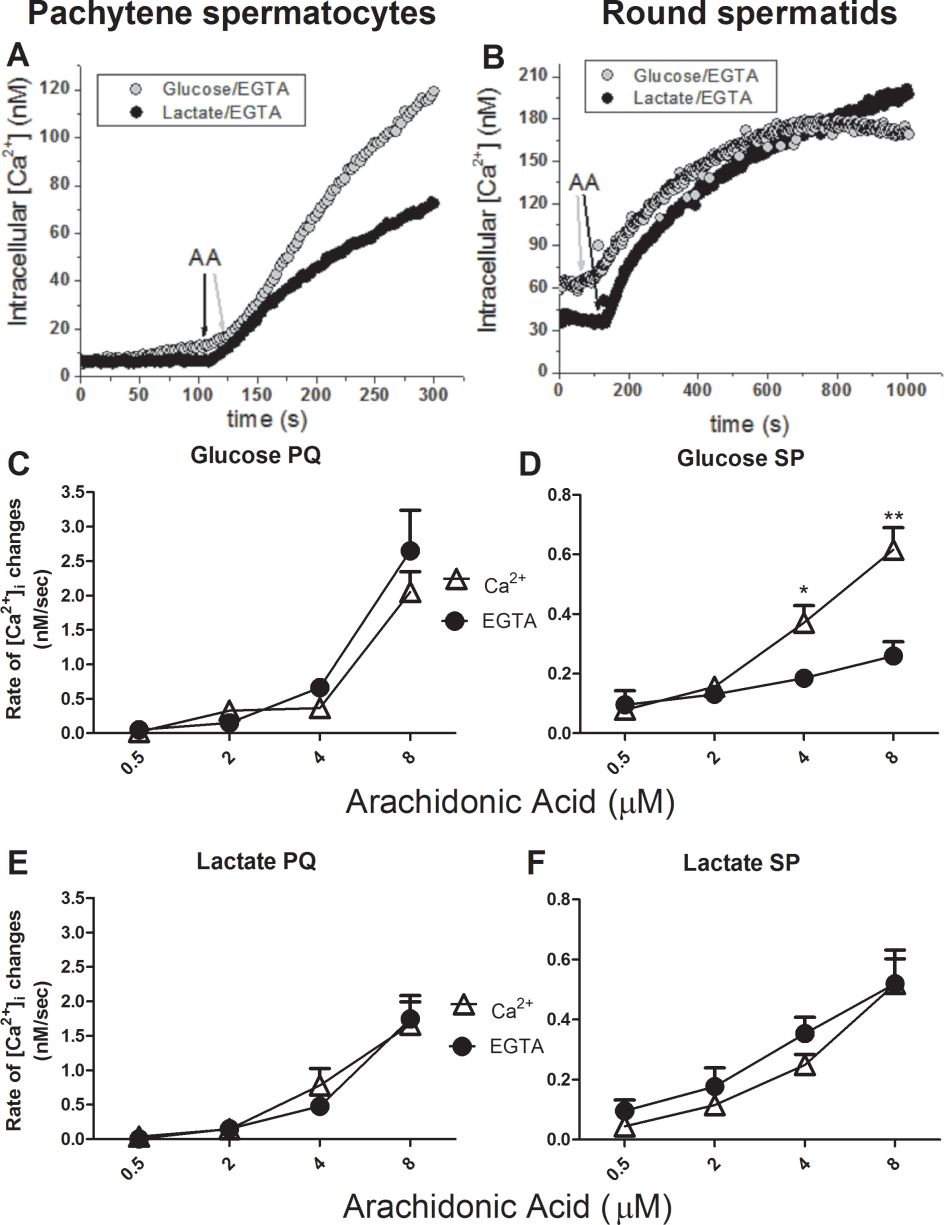
Metabolic substrates and external Ca^2+^ effects on AA-induced changes in [Ca^2+^]_i_. Dose response curves depicting the rate of [Ca^2+^]_i_ change (nM/s) vs. [AA] in the presence and absence of external Ca^2+^ and with 5 mM glucose or 5 mM L-lactate in the media surrounding suspensions of round spermatids **(A)** or pachytene spermatocytes **(B)**.

Because it was previously shown that Ca^2+^ homeostasis in isolated spermatogenic cells was dependent on which metabolic substrate was present in the media [41], we first tested the influence of AA on the [Ca^2+^]_i_ in pachytene spermatocytes or round spermatids incubated with glucose or lactate as metabolic substrates. In the presence of external lactate (Fig 3A and 3B), in both cell types there was an increase in initial rates of [Ca^2+^]_i_ changes that depended upon the external AA concentration but not the presence of external calcium. This extracellular calcium independence strongly suggests that AA was able to induce the release of Ca^2+^ from ICaS. A significant difference emerged in the presence of glucose, where the initial rise of [Ca^2+^]_i_ induced by AA was significantly higher in calcium-containing rather than in calcium-free media in round spermatids (Fig 3A). This difference is most likely due to the ability of glucose to release Ca^2+^ from the ICaS in round spermatids [8,41], a phenomenon associated to the ability of glucose to decrease intracellular [ATP] in round spermatids and a possible entry of external Ca^2+^ by activation of store-operated Ca^2+^ channels (Darszon et al., 2012). The glucose-dependent effect on initial rate of AA-induced [Ca^2+^]i rise observed in round spermatids was not observed in pachytene spermatocytes, which is in agreement with the known metabolic properties of these two cell types and their differences in [Ca^2+^]_i_ homeostasis [42].

As a positive control, cyclopiazonic acid (CPA), which can deplete the ICaS by inhibiting the sarcoplasm-endoplasmic reticulum Ca^2+^-ATPase (SERCA), was able to induce a rapid increase in [Ca^2+^]_i_ when added after AA in round spermatids and pachytene spermatocytes under all the conditions tested (with or without external Ca^2+^ and with lactate or glucose in each of those media; data not shown). These results demonstrate that the rates of [Ca^2+^]_i_ changes induced by UFAs were not limited by complete depletion of ICaS in the conditions tested in this work.

### Pharmacological testing for potential AA metabolism in the AA-induced [Ca^2+^]_i_ changes in round spermatids

**Fig 4:**
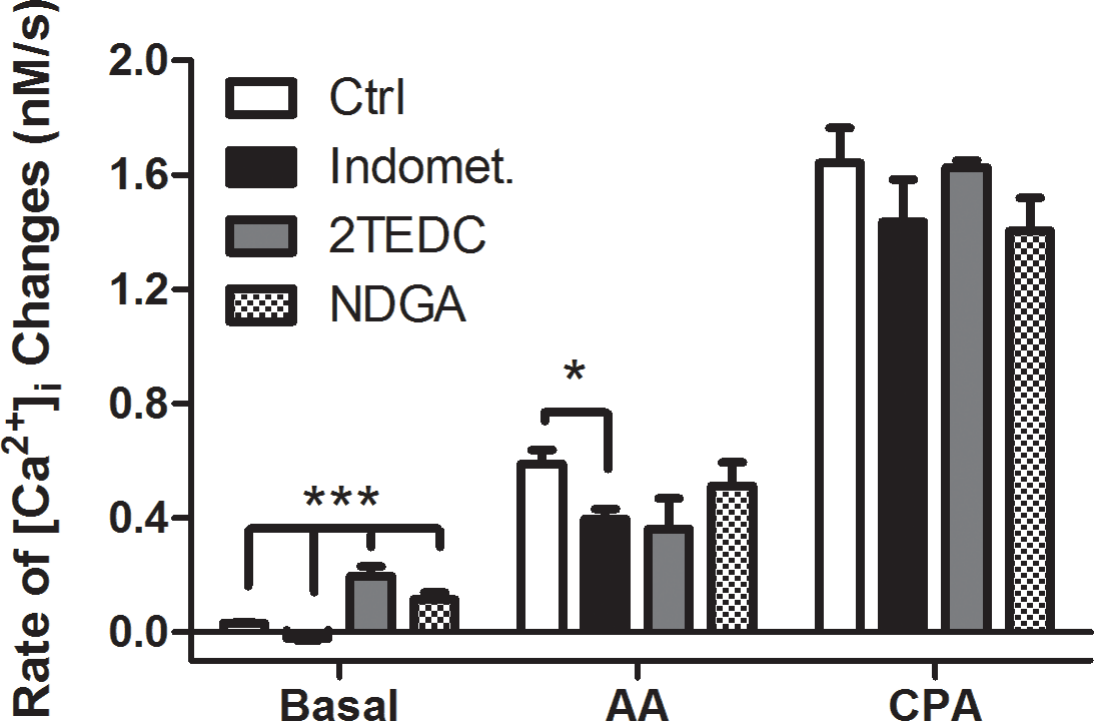
Inhibitors of AA metabolism do not affect AA-dependent [Ca^2+^]_i_ increases. Impact of inhibitors of AA metabolism (COX inhibitor: 10 μM indomethacin, and LOX inhibitors: 5 μM 2TEDC, 5 μM NDGA) on the AA-dependent and 2 μM cyclopiazonic acid dependent increase of [Ca^2+^]_i_ in KH-lactate media in the absence of external Ca^2+^. The Mann-Whitney statistical significance test was used to compare rates of [Ca^2+^]_i_ change with CPA vs. CPA-COX/LOX inhibitors, AA vs. AA-COX/LOX inhibitors and basal rate vs. COX/LOX inhibitors.

To determine whether AA and its effects on [Ca^2+^]_i_ could be mediated by cyclooxygenase (COX) or lipoxygenase (LOX) metabolites of AA, inhibitors of COX or LOX were tested (Fig 3). Our results demonstrate that the LOX inhibitor 2-TEDC (5 μM) was able to increase [Ca^2+^]_i_ on its own (in the absence of external Ca^2+^). The presence of the COX inhibitor indomethacin (10 μM), but not the LOX inhibitors NDGA (5 μM) or 2-TEDC (5 μM), induced a significant and partial reduction in the kinetics of the changes induced by 4 μM AA (33% decrease, Fig 4). Another AA metabolite, anandamide, was also tested in concentrations ranging from 2–8 μM (Fig 2). This endocanabinoid produced a small change in the rate of [Ca^2+^]_i_ increase that was only slightly superior to that generated by PA. By contrast, CPA induces a similar rate in the [Ca^2+^]_i_ change for all experimental groups. Taken together, these results strongly suggest that most of the AA-induced increase of [Ca^2+^]_i_ was not mediated by eicosanoid or endocannabinoid receptor activation after metabolic conversion of the AA offered to the cells.

### Effects of FAs on propidium iodide permeability and LDH release in spermatogenic cells

**Fig 5:**
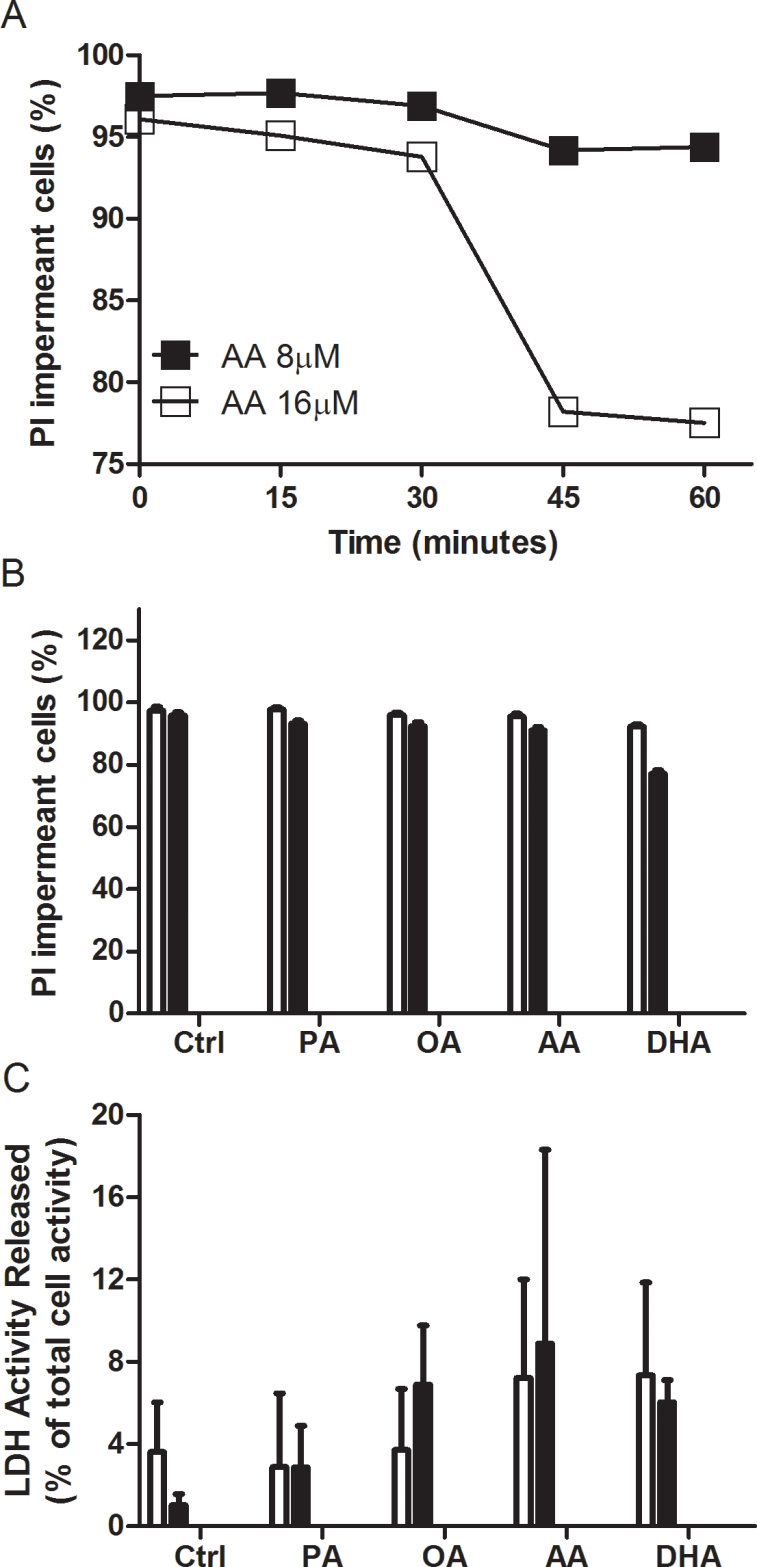
Effects of FA concentration and exposure time on the membrane integrity of round spermatids. **(A)** PI nuclear staining was used to monitor membrane integrity over time when 8 or 16 μM AA was applied to round spermatids. **(B)** PI nuclear staining was used to monitor membrane integrity following induction with 16 μM AA, DHA, OA and PA for 15 min (white bars) and 30 min (black bars) in round spermatids. **(C**) Percent release of intracellular LDH induced by 16 μM AA, DHA, OA and PA for 15 and 30 min in round spermatids.

The entry of propidium iodide (PI) into round spermatids was used to determine whether the FAs in this study affected germ cell integrity (Fig 5). AA was able to induce a significant increase in cell plasma membrane permeability at 16 but not at 8 μM in cells when incubated for more than 30 min (Fig 5A). Interestingly, at 16 μM none of the FAs studied were able to increase plasma membrane permeability in round spermatids after 15 min of incubation. Only 16 μM of DHA induced a significant increase in PI entrance to the cells after 30 min of incubation (Fig 5B). Considering the possibility that spermatids express connexin hemichannels through which PI can permeate [8,43], we next wanted to evaluate LDH leakage as a second criterion of plasma membrane permeability (Fig 5C). The results showed a high degree of variability but were similar to those of PI entry at both time points of FA exposure studied. These results indicate that the changes observed in [Ca^2+^]_i_ using lower FA concentrations and shorter exposure times (Figs 1–4) were not due to a non-specific FA-induced change in plasma membrane or inner membrane permeabilities.

### Effects of AA on ATP release in spermatogenic cells

Given that unsaturated FAs at > 8 μM concentrations were able to induce entry of PI into spermatogenic cells, and because PI can permeate connexin hemichannels and pannexin channels that play a role in ATP secretion (see [43,44]), we suspected that UFAs could activate these channels. If so, ATP would be released, which, in turn, could activate purinergic signalling in spermatogenic cells [45]. We tested whether ATP was released from spermatogenic cells in response to 15 μM AA. As shown in S1 Fig this was not the case. The ATP liberated from pachytene spermatocytes or round spermatids in response to this relatively high concentration of AA was less than 0.2% of the total ATP content in the cells and below the level of detection in our assay. Addition of 5–100 μM ATP or ADP did not produce significant changes in [Ca^2+^]_i_ in round spermatids or pachytene spermatocytes (data not shown).

### Effects of AA on plasma membrane potential

**Fig 6:**
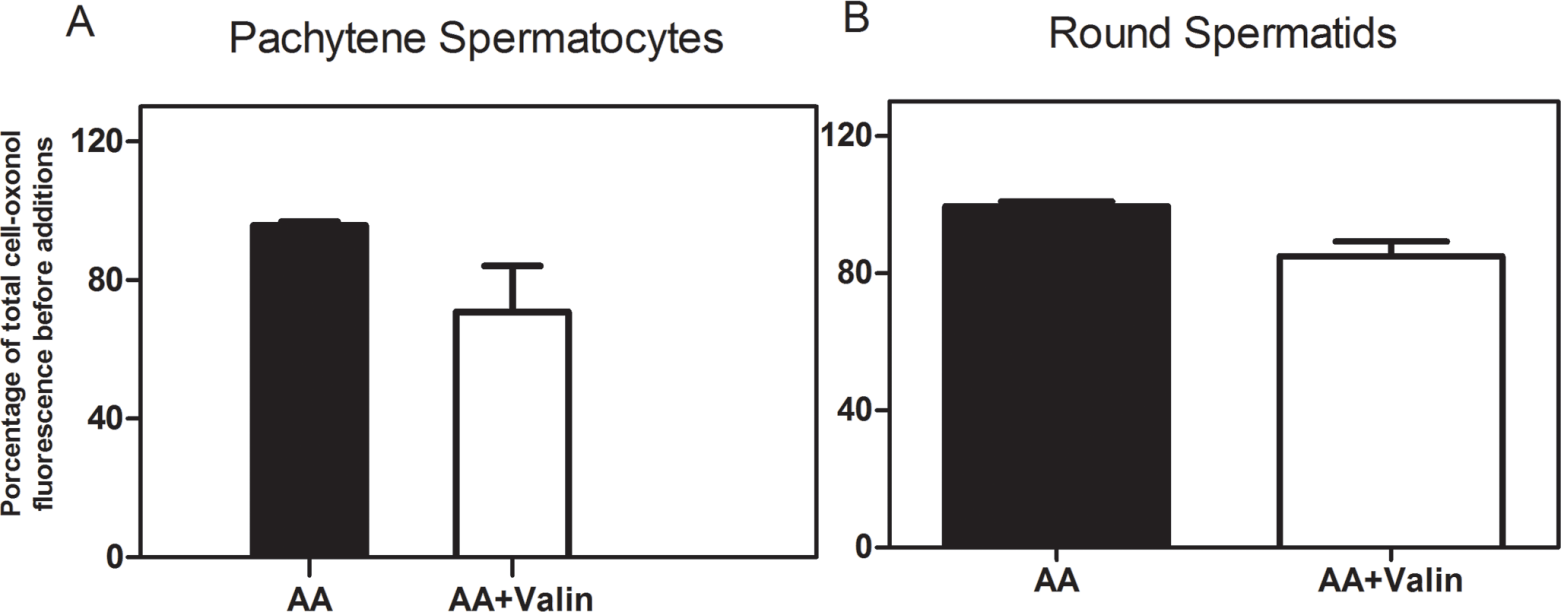
AA does not affect the plasma membrane potential in spermatogenic cells. Percent change in oxonol fluorescence induced by 4 μM AA with or without 0.5 μM valinomycin in pachytene spermatocytes **(A)** and round spermatids **(B)** in KH-lactate media in the absence of external Ca^2+^.

Although our results demonstrated that the changes in [Ca^2+^]_i_ induced by FA in spermatogenic cells were not associated with entry of Ca^2+^, the existence of a described voltage-dependence of some GPCRs [46] and voltage-sensing phosphoinositide phosphatases [47] prompted us to look for AA-induced changes in plasma membrane potential. The plasma membrane potential was estimated from the fluorescence of oxonol (Fig 6). An acute (5 min) addition of 4 μM AA did not produce significant changes in the oxonol fluorescence signal in pachytene spermatocytes or round spermatids resuspended in KH buffer, which strongly suggests that at concentrations of 4 μM or less this FA does not modify plasma membrane potential in these cells. As a positive control, valinomycin, a ionophore that increases K^+^ membrane permeability, induced a significant decrease in oxonol fluorescence when added after AA (Fig 6) in both pachytene spermatocytes and round spermatids, which indicates plasma membrane hyperpolarization [36].

### Effects of AA on intracellular pH

**Fig 7:**
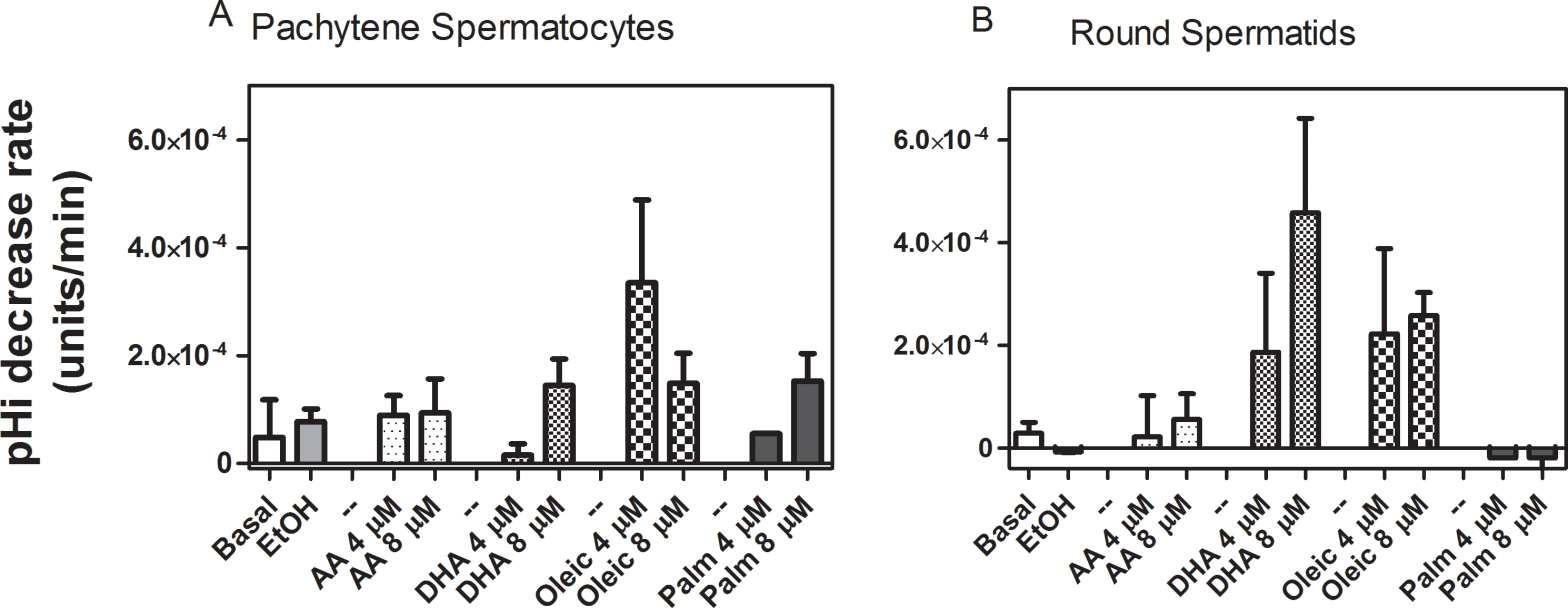
Effects of FA on pH_i_ changes. Spermatogenic cells, resuspended in KH media with no Ca^2+^, were exposed to 4 and 8 μM UFAs or PA. pH_i_ changes were monitored by BCECF fluorescence excitation ratio. Pachytene spermatocytes **(A)** and round spermatids **(B)** in KH-lactate media in the absence of external Ca^2+^.

Intracellular alkalinization can increase [Ca^2+^]_i_ [48] and intracellular acidification can decrease Ca^2+^ release from ICaS [49,50]. This trend prompted us to examine whether the FA-induced [Ca^2+^]_i_ changes we observe were really due to pH_i_ changes in spermatogenic cells. As shown in Fig 7, at a concentration of 4 μM none of the UFAs were able to induce a significant change in the pH_i_ of spermatogenic cells.

### Effects of FAs on spermatogenic cell mitochondrial membrane potential

**Fig 8:**
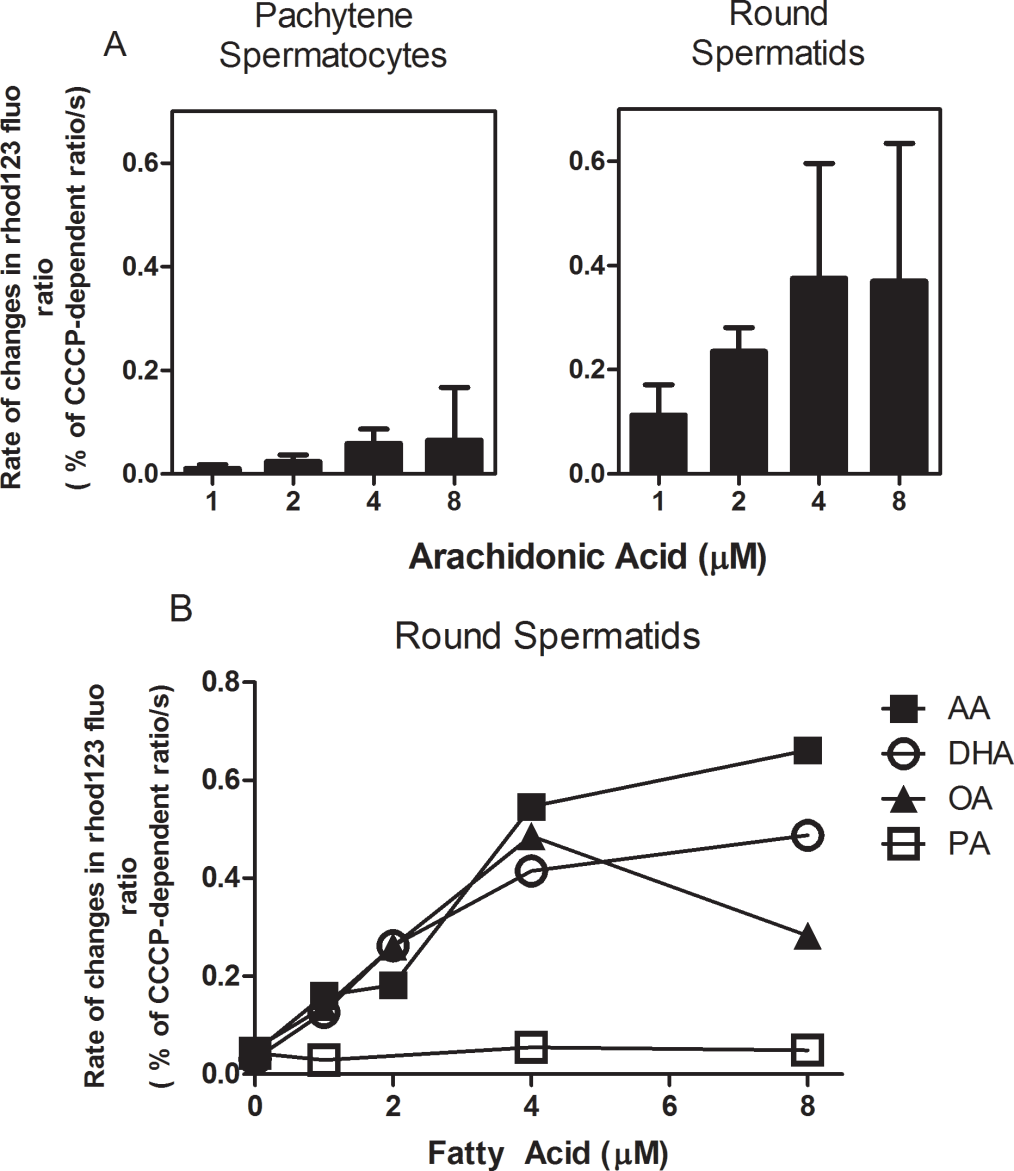
UFAs and PA have different effects on the mitochondrial membrane potential of spermatogenic cells. **(A)** Comparative effects of different concentrations of AA on mitochondrial membrane potential measured with rhodamine 123 fluorescence in pachytene spermatocytes and round spermatids cells permeabilized with digitonin. **(B)** Effects of different concentrations of UFAs and PA on round spermatid mitochondrial membrane potential measured with rhodamine 123 fluorescence in cells permeabilized with digitonin.

Free FAs are known to increase the permeability of mitochondrial inner membranes to H^+^, thereby inducing a decrease in mitochondrial inner membrane potential and mitochondrial uncoupling [49,51]. Mitochondria can be considered part of ICaS as they provide the ATP for Ca^2+^-dependent ATPases including SERCA. Therefore, our next question was whether FAs were able to decrease the mitochondrial membrane potential. This parameter was measured by rhodamine 123 fluorescence in permeabilized spermatogenic cells (Fig 8). AA, DHA and OA, but not PA, were able to induce a time-dependent decrease in the mitochondrial membrane potential, which strongly supports the idea that FAs cause a partial uncoupling of the mitochondrial oxidative phosphorylation (Fig 8A). The effects of AA at 1 μM, 2 μM, and 4 μM on the mitochondrial membrane potential were significantly lower in pachytene spermatocytes than in round spermatids (Fig 8B).

In spermatids, the slope of the mitochondrial membrane potential vs. FA concentration plot (Fig 8B) indicated that, with the exception of PA, the FAs examined were able to induce a drop in the mitochondrial membrane potential at 1 μM or higher concentrations. AA had the largest effect at all concentrations. Measurements with JC-1 in round spermatids with AA were consistent with these results (data not shown), strongly suggesting a partial mitochondrial uncoupling at 2 μM concentrations or higher.

### Effects of AA and DHA on ROS/RNS production in spermatogenic cells

As partial uncoupling of mitochondrial oxidative phosphorylation has been associated with decreased ROS production in this organelle [39,52], we next explored whether the uncoupling caused by UFAs in the mitochondria of round spermatids or pachytene spermatocytes was associated with changes in ROS production in these cells.

**Fig 9:**
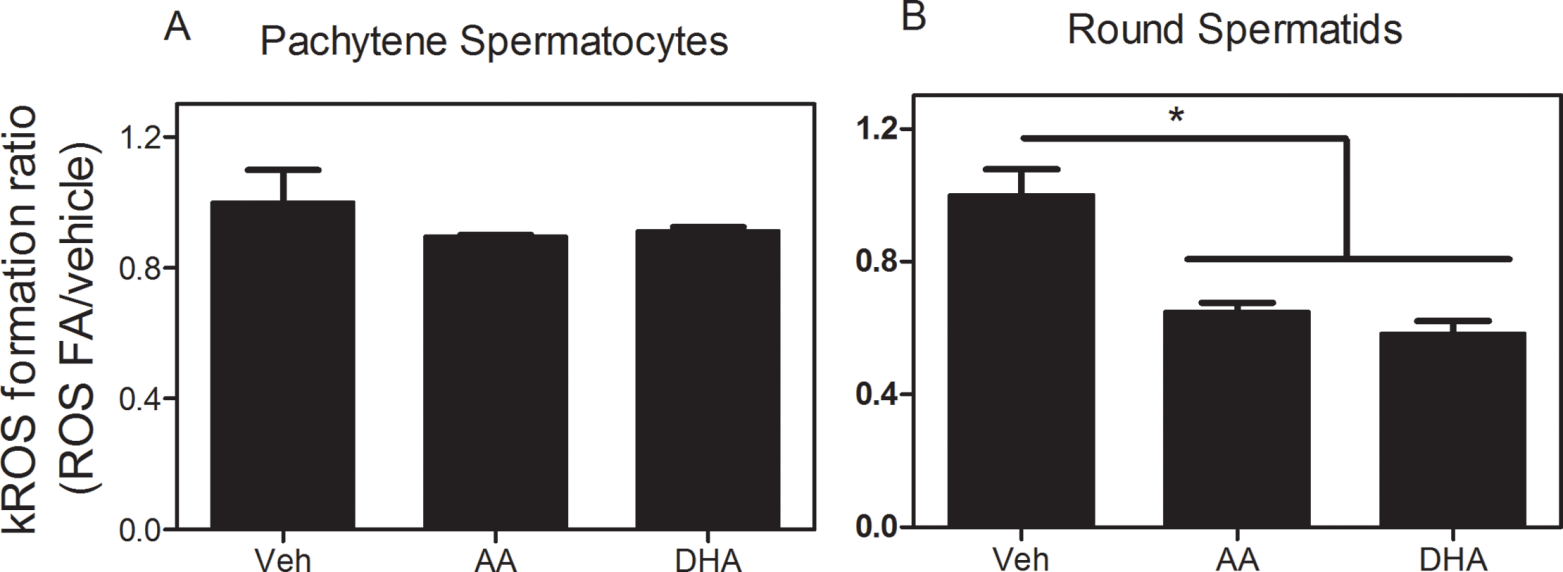
Effects of AA and DHA on the relative rate of ROS/RNS production by spermatogenic cells. At 4 μM of each FA the ROS/RNS levels were measured by the change in the fluorescence of H2DCFDA in pachytene spermatocytes **(A)** and round spermatids **(B)**.

At a concentration of 4 μM, the polyunsaturated FAs AA and DHA did not significantly modify the rate of ROS/RNS production in pachytene spermatocytes (Fig 9A), but induced a considerable decrease in round spermatids (Fig 9B). The cell-type dependent impact of these FAs on ROS/RNS production correlates well with their weaker effects on the mitochondrial membrane potential of spermatocytes relative to spermatids.

### Caspase 3 activation by AA in pachytene spermatocytes and round spermatids

At concentrations greater than 100 μM, AA can induce *in vitro* apoptosis in several cell lines [53–55]. Furthermore, [Ca^2+^]_i_ increase induced by AA has been associated with AA induction of cell apoptosis [56]. In order to determine if similar events were induced by AA in spermatogenic cells, we used caspase 3 activity as a criterion for apoptosis induction both in the absence and presence of external Ca^2+^.

**Fig 10:**
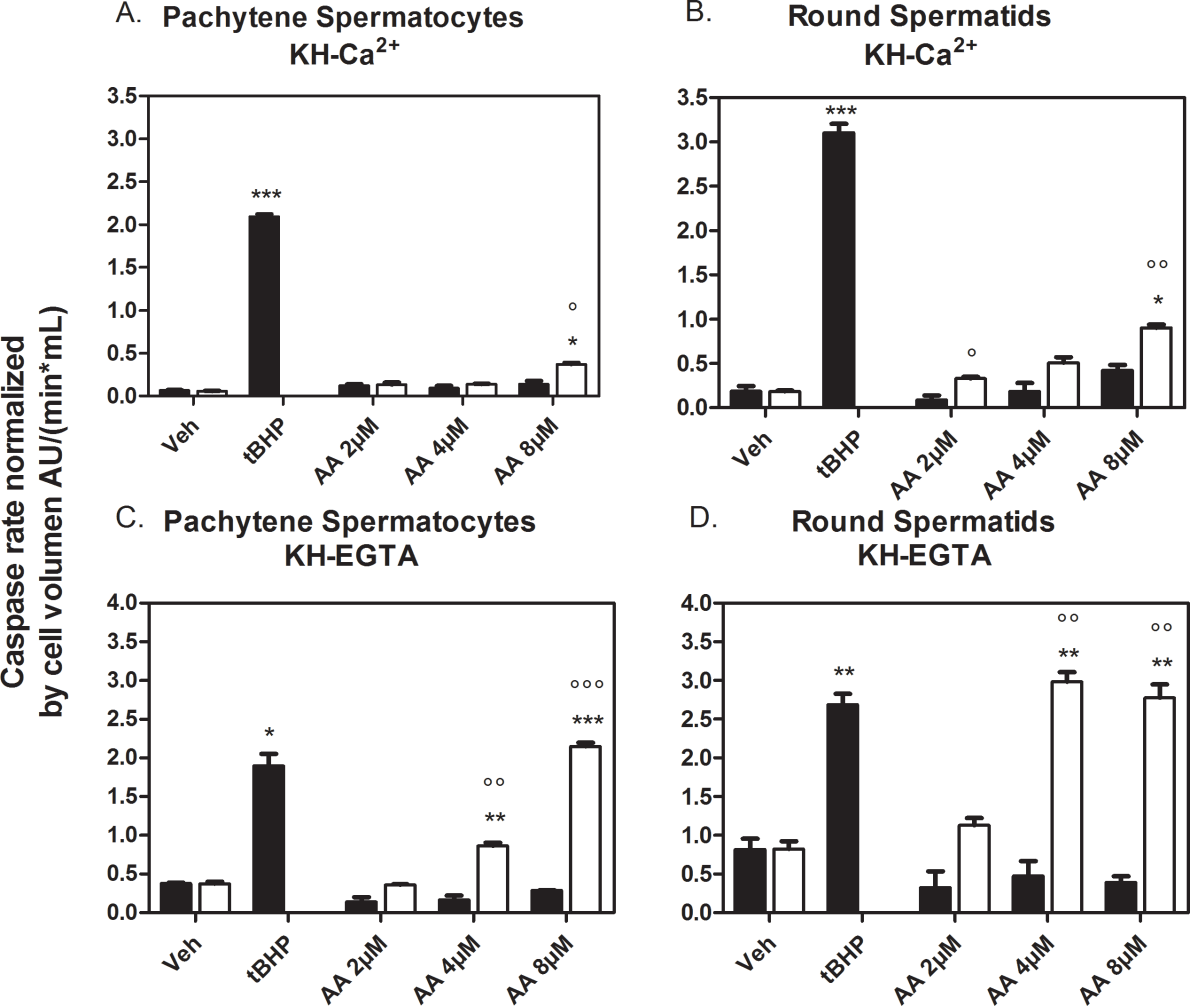
Effects of AA on caspase 3 activity in spermatogenic cells. Caspase 3 activity in the presence of external Ca^2+^ (0.5 mM) **(A,B)** and no Ca^2+-^EGTA **(C,D)** in pachytene spermatocytes (**A,C**) and round spermatids (**B,D**), respectively. Caspase 3 activity was measured in arbitrary fluorescence units per min and was normalized to the average cell volumes with diameters of 16 and 11 μm for pachytene spermatocytes and round spermatids, respectively. The final concentration of tBHP was 200 μM. Black bars are the average rates estimated between 0 and 60 min. White bars are the average rates estimated between 180 and 240 min. *Significance with respect to the vehicle. °Significance with respect to the 60 min time point at the same concentration. * or ° = p < 0.05, ** or °° = p < 0.01, *** or °°° = p < 0.001.

As a positive control, caspase 3 was activated in spermatogenic cells by tBHP with a lag time of approximately 10–20 min after tBHP addition (data not shown). tBHP activated caspase 3 in the absence and presence of external Ca^2+^ in both cell types. In calcium-free and calcium-containing media, AA-induced caspase 3 activation in spermatocytes and round spermatids but only after 180 min of treatment (Fig 10A and 10B). Interestingly, AA-induced caspase-3 activity at lower concentrations in round spermatids than in pachytene spermatocytes. The absence of external calcium (achieved with EGTA-containing media) caused spermatocytes and spermatids to be more sensitive to AA as it was able to induce caspase-3 at a lower concentration and to a higher extent than in a media with calcium (4 μM vs. 8 μM AA, Fig 9A and 9C). These results suggest that long-term exposures to AA in the seminiferous tubules could induce meiotic and post-meiotic germ cell apoptosis, and that the absence of external Ca^2+^ can sensitize spermatogenic cells to AA-induced long-term apoptosis.

## Discussion

The regulatory mechanisms that allow SC to control mammalian spermatogenesis are still poorly understood. It is known that SC release a number of soluble factors that could affect proliferation and differentiation of spermatogenic cells [1,57,58]. However, thus far very few of the molecules secreted by SC have been shown to functionally modify spermatogenic cells, especially meiotic and post-meiotic cells (see [59] for insulin-like growth factor II (IGF-I); [60] for activin; [8,9,41,42] for glucose and lactate; [61] for tumour growth factor β (TGFβ). One of the molecules secreted by SC and regulated by FSH is AA [10]. The results of the present study demonstrate that AA, and other UFAs, are able to induce a significant Ca^2+^ release from ICaS in germ cells. This UFA-dependent [Ca^2+^]_i_ rise was not mediated by a loss of membrane integrity, by changes in plasma membrane potential, or by activation of ATP release from spermatogenic cells.

Increases in [Ca^2+^]_i_ were apparently specific to UFAs, as shown by the lack of effect of PA and by the different responses of spermatids to AA and DHA. The effect of OA was intermediate between PA and polyunsaturated FA, as higher concentrations of OA were required to increase [Ca^2+^]_i_ to levels similar to those induced by AA or DHA. The effects may be ascribed directly to the fatty acids rather than to some of their bioactive metabolites, as shown in the case of AA by the negligible effect of adding anandamide (an AA-derived endo-cannabinoid metabolite) in similar concentrations as AA to the cells, or by pharmacologically blocking the conversion of AA via cyclooxygenase or lipoxygenase inhibitors.

Rhodamine 123 in cells permeabilized with digitonin allowed us to determine the effects of UFAs on the mitochondrial membrane potential. Effects were dependent on cell type and FA species. The ability of UFAs to induce a partial decrease in membrane potential was weaker in pachytene spermatocytes than in round spermatids. In the latter, the magnitude of the UFA-induced decrease in the mitochondrial membrane potential was consistent with what has been described as mild uncoupling [51], and thereby would be expected to decrease the efficiency of mitochondrial ATP synthesis. By measuring intracellular adenine nucleotides, a glucose induced decrease in ATP levels was previously observed to occur in round spermatids to a greater extent than in pachytene spermatocytes due to differences in energy metabolism [42]. As these decreases occurred concomitantly with increases in [Ca^2+^]_i_ they were ascribed to the ATP-dependent maintenance of Ca^2+^ homeostasis. This agrees with the results of the present study, in which UFA, in spermatids more so than in spermatocytes, induces a relatively monotonic increase in the rate of Ca^2+^ release from ICaS and a concomitant decrease in mitochondrial membrane potential as concentrations of UFAs increase.

The different uncoupling effects exerted by UFA on the mitochondria of each of these spermatogenic cell types is possibly linked to the observation that AA and DHA were able to decrease ROS production in round spermatids but not in pachytene spermatocytes. The ability to be partially uncoupled by several stimuli [39] is emerging as an interesting and differential aspect of round spermatid physiology.

The kinetics of AA-induced caspase 3 activation (occurring after 180 min of treatment) demonstrated that this was a late event as compared to AA-induced intracellular Ca^2+^ release. The fact that caspase 3 activation was more prominent in the absence of external Ca^2+^ strongly suggests that these cells had shifted to a pro-apoptotic state, likely due to the lowering of ICaS.

Because AA can be released by SC in a FSH-dependent manner [10], and probably other UFAs as well, our results demonstrate that as free FAs, UFAs can modify [Ca^2+^]_i_ and exhibit different effects on mitochondrial function in pachytene spermatocytes and round spermatids. These results strongly suggest that FAs originating in SC could be part of the cell-cell signalling mechanisms that control or modulate spermatogenic cell functions like metabolism, proliferation, differentiation or death in the seminiferous epithelium.

## Supporting Information

S1 Fig Effects of AA on the extracellular luciferin-luciferase luminescence over time. **(A)** Pachytene spermatocytes or **(B)** round spermatids were exposed to 8 μM AA and subsequently to 25 μg/ml digitonin.
(TIF)
