# Supplementary figures and images for: Effects of Fatty Acids on Intracellular [Ca2+], Mitochondrial Uncoupling and Apoptosis in Rat Pachytene Spermatocytes and Round Spermatids
Source: PLoS One. 2016 Jul 18;11(7):e0158518. doi: 10.1371/journal.pone.0158518 (PMC4948913; doi:10.1371/journal.pone.0158518)

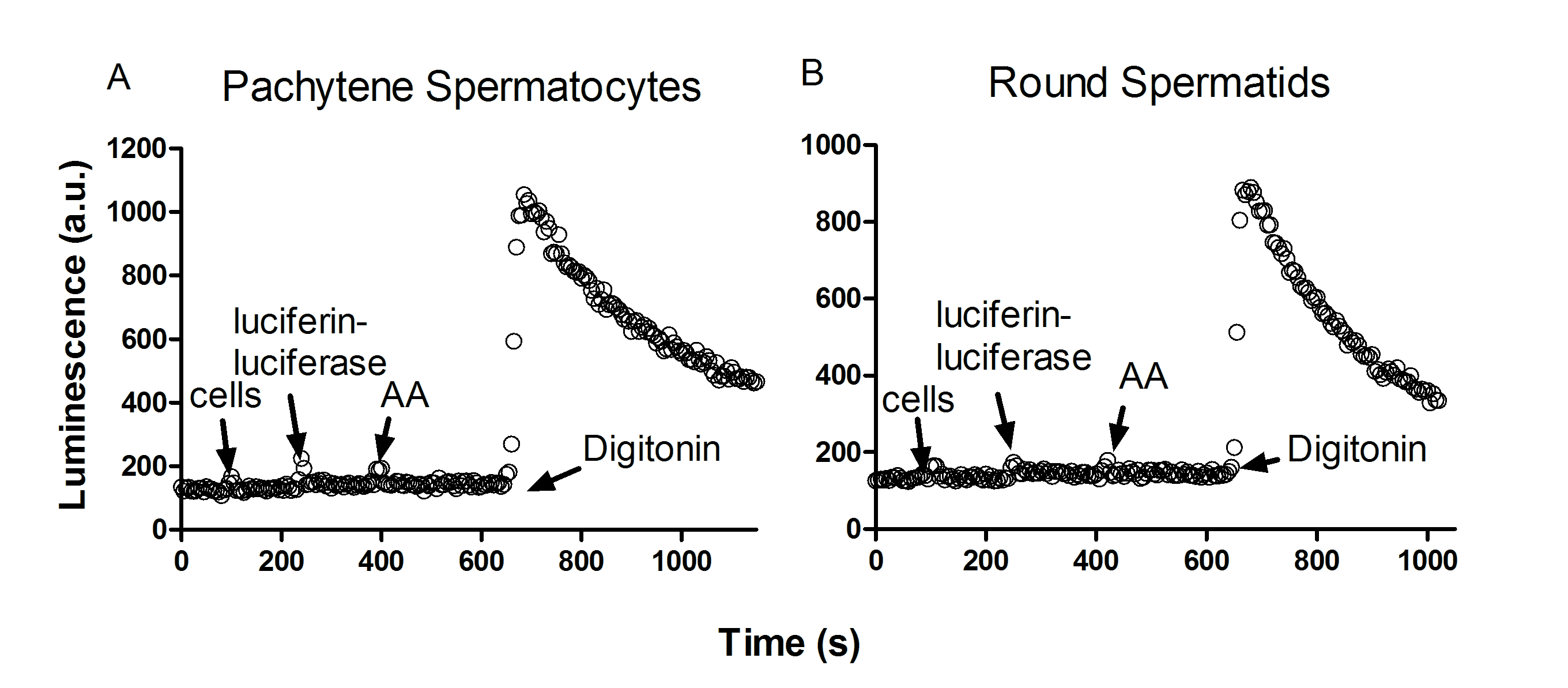

Supplement: S1 Fig — (A) Pachytene spermatocytes or (B) round spermatids were exposed to 8 μM AA and subsequently to 25 μg/ml digitonin. (TIF) [file pone.0158518.s001.tif]
